# HDL particles incorporate into lipid bilayers – a combined AFM and single molecule fluorescence microscopy study

**DOI:** 10.1038/s41598-017-15949-7

**Published:** 2017-11-21

**Authors:** Birgit Plochberger, Clemens Röhrl, Johannes Preiner, Christian Rankl, Mario Brameshuber, Josef Madl, Robert Bittman, Robert Ros, Erdinc Sezgin, Christian Eggeling, Peter Hinterdorfer, Herbert Stangl, Gerhard J. Schütz

**Affiliations:** 1 TU Wien, Institute of Applied Physics, Vienna, 1040 Austria; 2 Johannes Kepler University Linz, Institute of Biophysics, Linz, 4020 Austria; 3 Upper Austria University of Applied Sciences, Campus Linz, Linz, 4020 Austria; 4 Medical University of Vienna, Center for Pathobiochemistry and Genetics, Institute of Medical Chemistry, Vienna, 1090 Austria; 5 Center for Advanced Bioanalysis GmbH, Linz, 4020 Austria; 6 Keysight Technologies Austria, Linz, 4020 Austria; 7 Queens College of the City University of New York, Department of Chemistry and Biochemistry, Flushing, NY 11367 USA; 8 Arizona State University, Department of Physics, Tempe, AZ 85287-1504 USA; 9 Max Planck Institute of Molecular Cell Biology and Genetics, Dresden, 01307 Germany; 10 MRC Human Immunology Unit, Weatherall Institute of Molecular Medicine, University of Oxford, Oxford, OX3 9DS UK

## Abstract

The process, how lipids are removed from the circulation and transferred from high density lipoprotein (HDL) – a main carrier of cholesterol in the blood stream – to cells, is highly complex. HDL particles are captured from the blood stream by the scavenger receptor, class B, type I (SR-BI), the so-called HDL receptor. The details in subsequent lipid-transfer process, however, have not yet been completely understood. The transfer has been proposed to occur directly at the cell surface across an unstirred water layer, via a hydrophobic channel in the receptor, or after HDL endocytosis. The role of the target lipid membrane for the transfer process, however, has largely been overlooked. Here, we studied at the single molecule level how HDL particles interact with synthetic lipid membranes. Using (high-speed) atomic force microscopy and fluorescence correlation spectroscopy (FCS) we found out that, upon contact with the membrane, HDL becomes integrated into the lipid bilayer. Combined force and single molecule fluorescence microscopy allowed us to directly monitor the transfer process of fluorescently labelled amphiphilic lipid probe from HDL particles to the lipid bilayer upon contact.

## Introduction

Proper supply of mammalian cells with cholesterol is crucial for membrane function and cellular survival and is regulated by several mechanisms, including cellular uptake, synthesis, storage and export^1^. Particularly, cholesterol plays an important role in regulating membrane fluidity and elasticity^2^. Transport of cholesterol in the blood stream is facilitated by lipoproteins, specialized cargo vehicles made of a flexible lipophilic protein scaffold that can adapt to different loads of lipid cargo. The exchange of lipids between lipoproteins and cells is a key process for maintaining cellular cholesterol homeostasis. Thus, imbalance of cholesterol uptake and export leads to cardiovascular disorders such as atherosclerosis^3^, diabetes^4^ and cancer^5^.

In particular, the transport of excess cholesterol from the periphery back to the liver for excretion into the bile is achieved by high-density lipoproteins (HDL)^6^. After transport through the circulation, cholesterol has to be transferred from HDL particles to hepatocytes for disposal. In depth knowledge on the mechanism about how lipoproteins exchange lipids with cell membranes is therefore a prerequisite for understanding and treating lipid disorders. Overall, this transport, called reverse cholesterol transport (RCT), is a highly regulated process that relies on specific interactions between HDL particles and cell membranes^6^. It involves multiple steps and components, such as cholesterol efflux from lipid-laden macrophages, HDL-modifying enzymes, and hepatic HDL receptors.

Together with triglycerides, cholesteryl esters (CEs) are transported in the hydrophobic core of HDL particles; free cholesterol, phospholipids, and apolipoproteins build up the particles’ amphiphilic surface monolayer. The amphipathic α-helices of the main HDL protein apolipoprotein A-I (apoA-I)^7,8^ are embedded on the surface of HDL, just above the head group region of the phospholipids^9^. Pronounced plasticity indicates that apoA-I can undergo substantial conformational changes^8^. Of note, N- and C-terminal α-helical regions of apoA-I were found to be partly pushed away from oil-water interfaces^10^, suggesting that parts of apoA-I may transiently protrude from the HDL surface. ApoA-I is also the major binding factor by which the scavenger receptor, class B, type I (SR-BI), the so-called HDL receptor, captures the HDL particle from the blood stream and thereby tethers it to the plasma membrane of the target cell^11^. The details of subsequent cholesterol transfer at the target cell membrane, however, are still controversially discussed.

In contrast to the well-described uptake of low density lipoprotein (LDL) by the liver, in which the whole particle is endocytosed and degraded, cargo transfer via HDL proceeds without particle degradation; this uptake process is termed selective CE uptake^12,13^. The lipid transfer process itself, however, is unclear: it has been proposed to occur after HDL endocytosis^14,15^, or directly at the cell surface across an unstirred water layer or via a hydrophobic channel in the receptor^16–18^. Interestingly, on model systems cargo transfer between synthetic lipid bilayers and HDL was observed^19^, indicating a role of the target membrane itself on the lipid uptake process.

In this work we focused on the first step of HDL-mediated lipid transfer, the docking of the HDL particle itself and the lipid transfer process immediately after docking. We used high-end single molecule microscopy and atomic force microscopy, which allowed us to directly visualize the interaction of the particle with the cell surface or synthetic lipid bilayers. Upon contact, we observed HDL being incorporated into the hydrophobic core of lipid bilayers, and amphiphilic cargo being released immediately.

## Results

**Figure 1:**
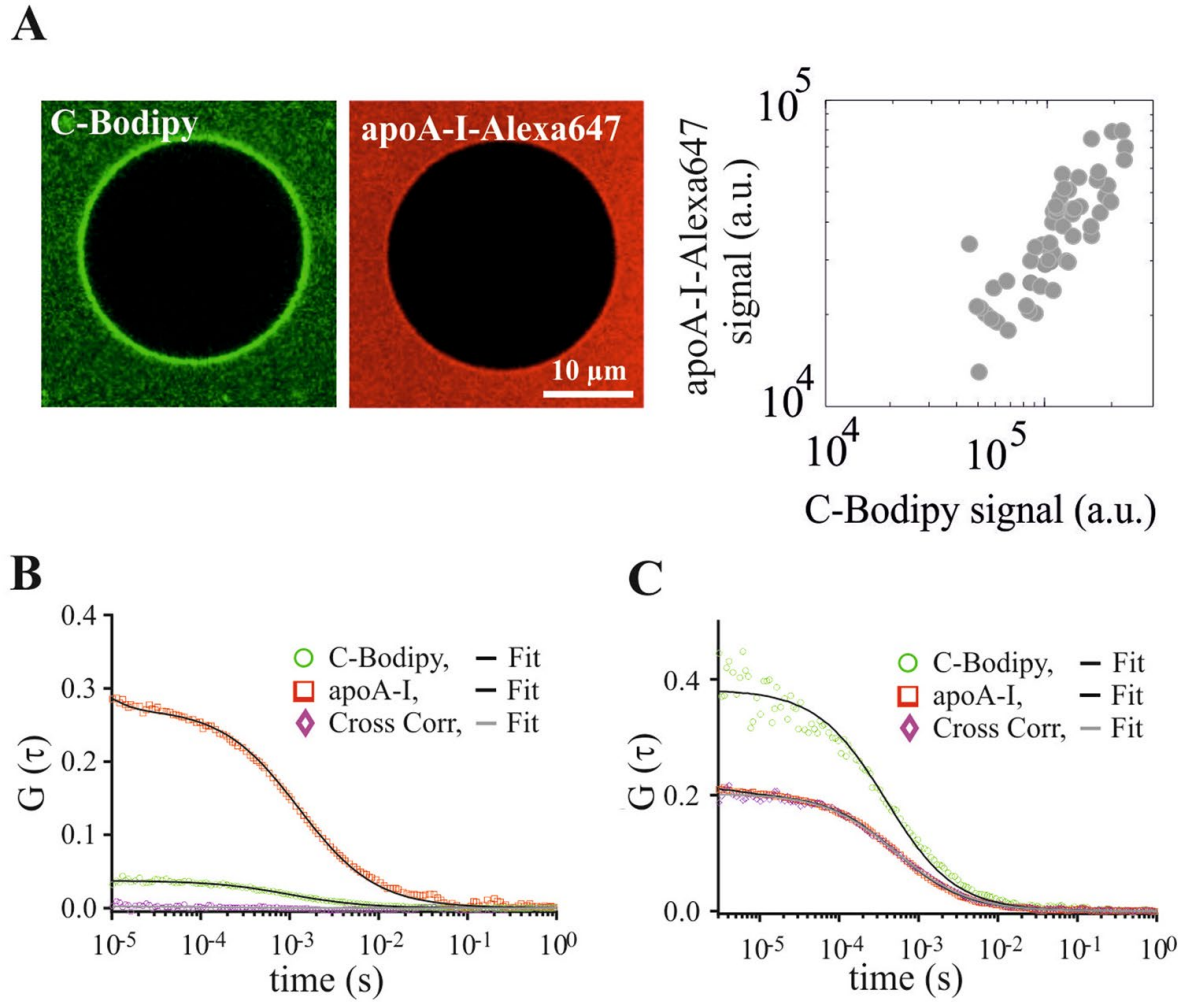
GUVs made of DOPC were incubated with fluorescently labelled HDL. (**A**) Representative images of a single GUV in the C-Bodipy channel (green) and the apoA-I-Alexa647 channel (red) are shown (left). The right panel shows the C-Bodipy versus the apoA-I-Alexa647 signal for single vesicles. (**B**,**C**) FCS and FCCS on apoA-I-Alexa647 and C-Bodipy. Shown are FCS and FCCS curves for the indicated probe molecules after fusion of HDL particles with GUV membranes (**B**) or in buffer solution (**C**). Panel (C) shows high cross-correlation when HDL particles were intact (red and pink curves virtually identical) whereas panel (B) shows no notable cross-correlation after HDL fusion with the DOPC membrane. Black solid lines represent the fits.

We first studied the interaction of HDL with fluid lipid bilayers by confocal microscopy. For this, we used HDL particles containing Alexa647-labelled lipoprotein and Bodipy-labelled cholesterol (C-Bodipy). After addition of the fluorescence-labeled HDL to giant unilamellar vesicles (GUVs), we observed a clear membrane stain in the C-Bodipy channel, and a faint membrane stain in the apoA-I-Alexa647 channel (Fig. 1A; see also Fig. S1). Differences in the contrast arise from the much higher number of C-Bodipy molecules per HDL particle compared to apoA-I-Alexa647. Quantitative analysis of the apoA-I-Alexa647 and C-Bodipy signals revealed a strong correlation (Fig. 1A, PEARSON correlation coefficient = 0.91), confirming transfer of both cholesterol and apoA-I.

Additionally, fluorescence correlation spectroscopy (FCS) experiments indicate that the C-Bodipy was freely mobile in the GUV membrane (Fig. 1B, green curve), and also apoA-I-Alexa647 showed mobility, (Fig. 1B, red curve). Generally, the amplitude of an FCS curve is inversely proportional to the number of molecules within the confocal volume. In panel (B), the amplitude for C-Bodipy is significantly lower than apoA-I-Alexa647 confirming the higher number of C-Bodipy particles compared to apoA-I-Alexa647 in the observed GUV membrane. In contrast, the amplitudes are similar in panel (C), as the intact HDL particle diffuses through the confocal volume. The difference between the amplitudes in (C) is due to a smaller focal spot for 488 nm excitation compared to 640 nm excitation.

In order to study the separation of C-Bodipy from apoA-I-Alexa647, we performed two color fluorescence cross-correlation spectroscopy (FCCS): for the GUV membrane, zero cross-correlation amplitude revealed the absence of co-diffusion of the two species (Fig. 1B magenta), indicating that cargo was transferred and separated from the particle. In contrast, HDL in buffer showed clear co-diffusion of apoA-I-Alexa647 and C-Bodipy (Fig. 1C).

**Figure 2:**
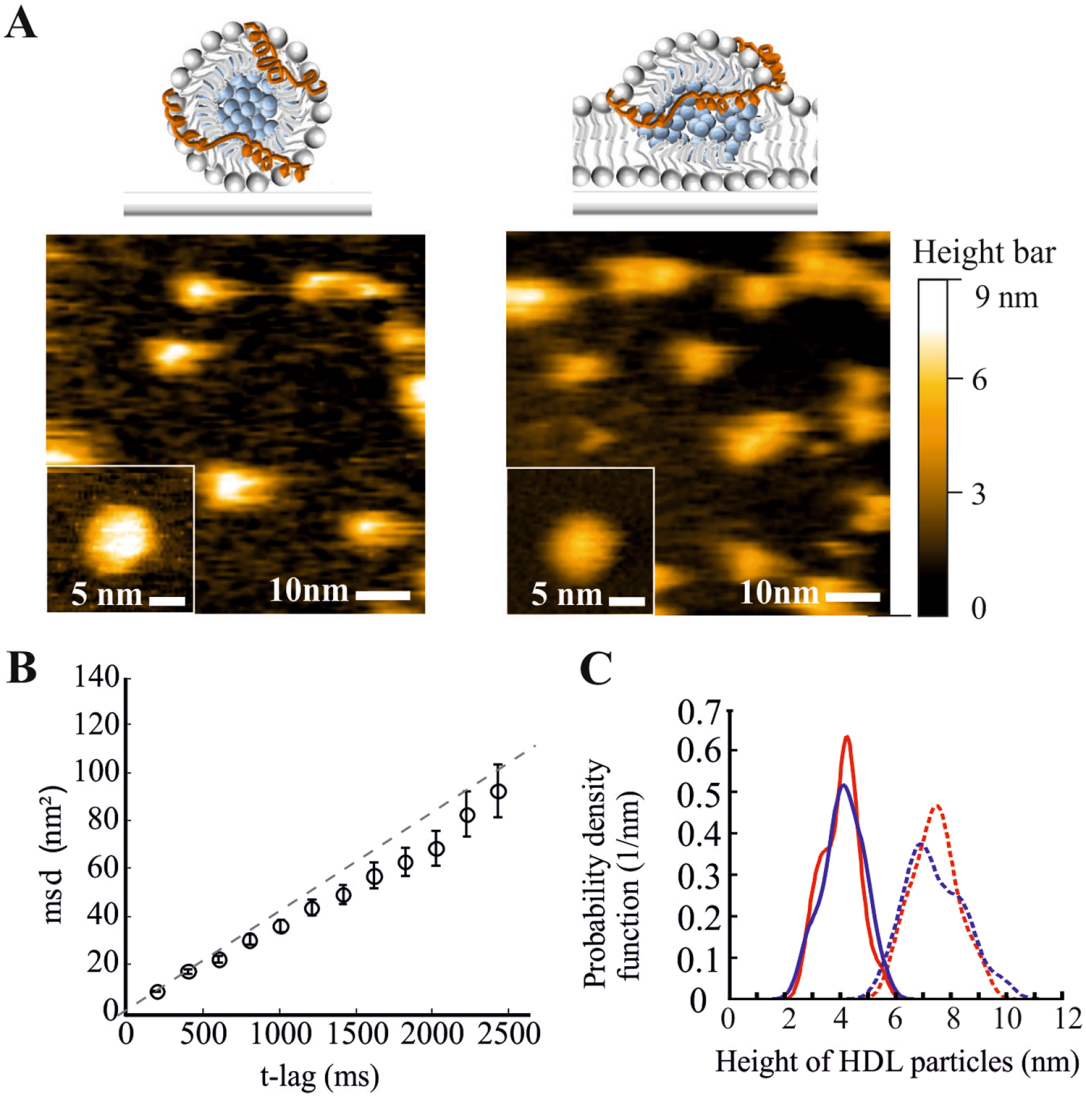
HDL incorporates into supported lipid bilayers. (**A**) High-speed AFM images of native HDL on mica (**left**) and on a supported DOPC bilayer (**right image**). (**B**) Diffusion analysis of added HDL particles on supported DOPC bilayers. The plot shows the mean-square displacement versus time for inserted HDL particles (20 particles, frame rate = 12–20 frames per second). Diffusion was analysed with Eq. 2, yielding a diffusion constant of D = 10.44 ± 1.2 nm^2^/s. Error bars indicate the standard error of the mean. (**C**) Height analysis of native HDL particles (red) and reconstituted HDL particles (blue) adsorbed to mica (dashed lines) or DOPC bilayers (full lines). The histograms reveal two distinct peaks: the peak at 7.4 nm corresponds to the size of HDL particles, the peak at 4.0 nm to the height of particles incorporated into the lipid bilayer.

To explore the molecular details of the interaction between HDL particles and biomembranes at nanometer resolution we applied atomic force microscopy (AFM). Reconstituted or native HDL particles (rHDL or nHDL, respectively) were added to a mica-supported fluid DOPC bilayer, or immobilized directly on the mica surface. For imaging we used a novel high-speed AFM^20^, which allows for imaging macromolecular dynamics^21^ and interactions^22^ at negligible forces with nanometer spatial and subsecond temporal resolution. We observed individual HDL particles as isolated protrusions from the flat surface (Fig. 2A). While HDL was immobile on the mica surface, it showed low but significant mobility on the DOPC bilayer (Movie S1): single particle tracking analysis revealed a lateral diffusion constant D = 10.44 ± 1.22 nm^2^/s (Fig. 2B).

On mica, spherical HDL particles were observed with a height of 7.4 nm (Fig. 2C) and a width ranging between 6.5 and 9 nm (Fig. S2). When HDL particles were added to the supported DOPC bilayer, the average height was significantly reduced to 4.0 nm (Fig. 2C), indicating incorporation of HDL into the bilayer. Similar results were obtained for rHDL and nHDL.

**Figure 3:**
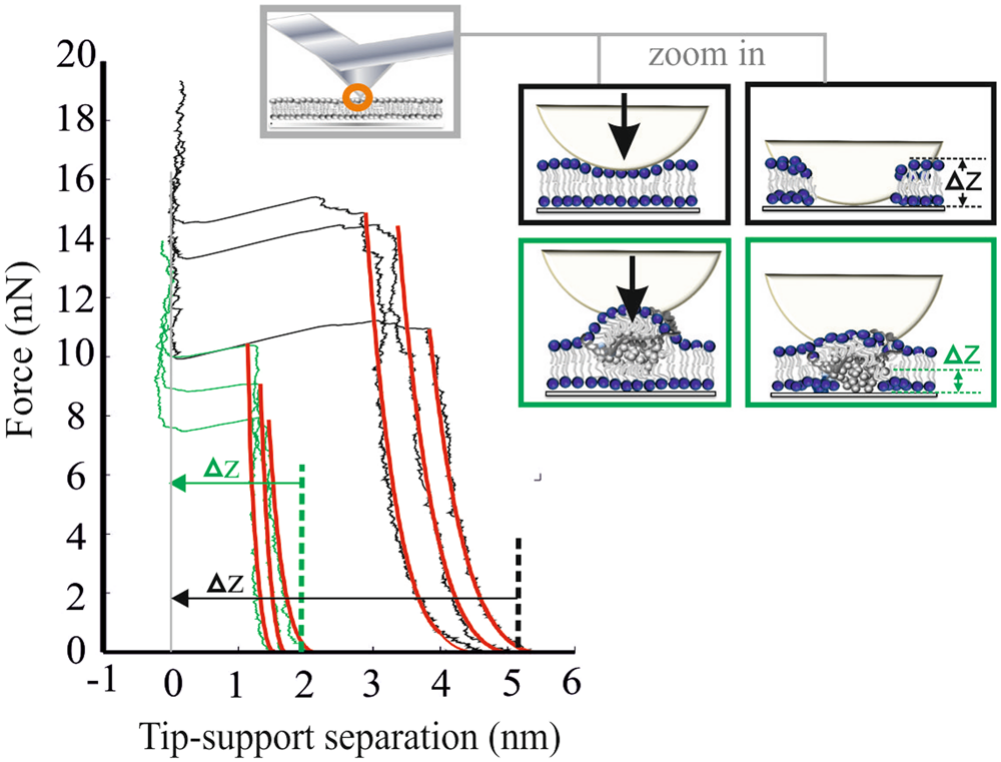
AFM indentation experiments reveal leaflet penetration of HDL particles. Experiments were performed on mica-supported DOPC bilayers using bare silicon tips (black) or HDL-coated tips (green). Representative approach curves are shown upon contact with the bilayer. Elastic bilayer deformation below the breakthrough force F_p_ was fitted with Eq. 1 (red). The intersection with the x-axis gives the thickness of the penetrated membrane layer (dashed lines), yielding 5.2 ± 0.8 nm (uncoated tips) and 2.0 ± 0.4 nm (HDL-coated tips). See also Fig. S3.

Additionally, experiments designed to monitor leaflet penetration by HDL particles were conducted. For this, supported lipid bilayers were penetrated with bare or HDL-coated AFM tips and the obtained approach curves were analyzed. Under both conditions we found force-distance curves of similar shape: upon contact, the tip compressed the membrane elastically, followed by abrupt penetration at a force F_p_, typically in the ten Nanonewton range (Fig. 3 and Fig. S3). For each approach curve we determined the point of the first counter-force acting on the tip, and calculated the corresponding distance from the glass surface Δz. Bare tips yielded distances of Δz = 5.2 ± 0.8 nm (black lines), whereas tip-coating with HDL reduced the distance to Δz = 2.0 ± 0.4 nm (green). Force spectroscopy thus reveals that HDL-tips penetrate deeper into the bilayer core before experiencing a counter force. Together the data support the view that HDL particles incorporate into the supported lipid bilayer upon contact.

**Figure 4:**
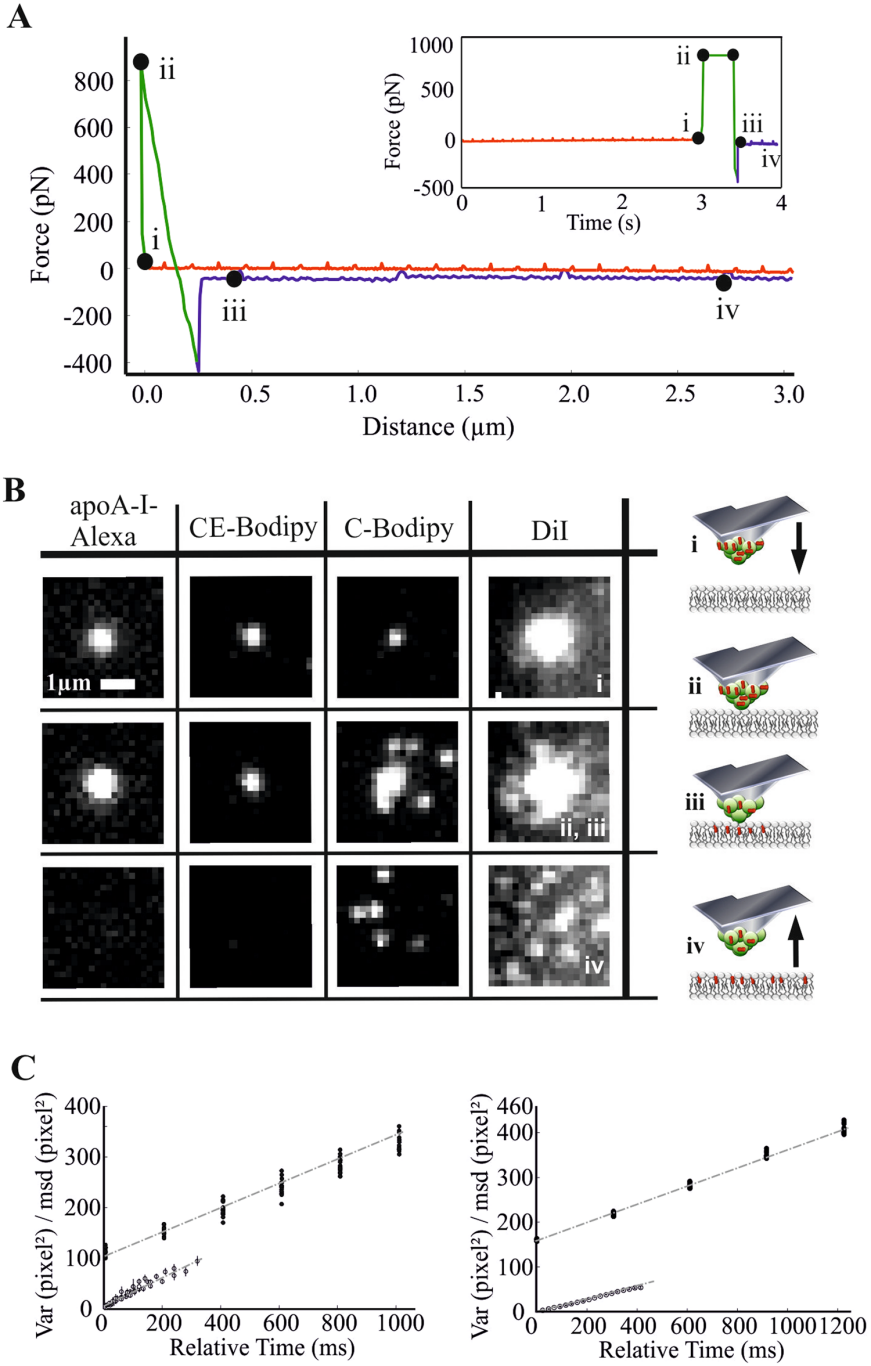
Synchronized AFM and single molecule fluorescence microscopy experiments. (**A**) Representative force curve of a transfer experiment. AFM-tips were functionalized with fluorescent HDL and brought into contact with glass-supported DOPC bilayers (i), kept at constant force F < F_p_ for 500 ms (ii-iii), and finally retracted (iv). The small spikes in the trace and retrace curve are caused by the excitation laser, which is partially detected on the photodiode of the AFM. (**B**) Fluorescence images at the indicated time points are shown for HDL pre-loaded with C-Bodipy, DiI, or CE-Bodipy; for control, also the apoA-I-Alexa647 signal is shown. Upon contact, only C-Bodipy and DiI – but not CE-Bodipy and the covalently linked apoA-I-Alexa647 – moved away from the contact point and diffused freely in the bilayer after tip retraction. Diffusion analysis of transferred versus pre-inserted C-Bodipy (**C**, **left**) and DiI (**C**, **right**) in supported DOPC bilayers. The plot shows the time-dependent variance for transfer experiments (filled circles), and the mean-square displacements versus time for pre-incubated samples (open circles). For transfer experiments, the time-point zero specifies the first image, after the AFM-tip was retracted from the surface. Experiments were fit with Eq. 2 (pre-incubated samples) or Eq. 3 (transfer experiments). From the slopes of the C-Bodipy data (**left**), we calculated a diffusion constant of D = 2.9 ± 0.29 µm^2^/s and D = 3.3 ± 0.87 µm^2^/s for the transfer experiments and the pre-incubated sample, respectively. The same analysis was performed for DiI (**right**), yielding D = 1.36 ± 0.04 µm²/s for the transferred probe and D = 1.06 ± 0.02 µm²/s for the pre-incubated probe. Error bars indicate the standard error of the mean.

To substantiate that particle incorporation is a prerequisite for cargo exchange, we developed a combined microscopy system, with which we could deliver HDL particles to supported membranes in a highly controlled way via the AFM tip, while simultaneously recording the transfer of the fluorescent probe via single molecule fluorescence microscopy in total internal refection- (TIR-) mode. For this, we reconstituted HDL with fluorescent lipids representing the main lipid classes of HDL (free cholesterol, esterified cholesterol and diacyl-lipids). AFM-tips coated with fluorescent rHDL were brought into contact with supported lipid bilayers up to a maximum force F < F_p_, so that the particles were actually incorporated into the membrane without rupturing the bottom leaflet (Fig. 4A). Lowering the tip to a distance of 20–50 nm from the bilayer yielded no fluorescence signal in the membrane, signifying that without actual contact, labeled lipids could not cross the hydrophilic barrier between the particle and the membrane. Within a few milliseconds after contact of the tip and the membrane, however, single fluorescent cargo molecules were observed to diffuse out of the contact region in case of BODIPY-cholesterol^23^ (C-Bodipy, representing free cholesterol) and the neutral lipophilic dye DiI (Movie S2); no diffusing signals were observed for the hydrophobic cargo BODIPY-cholesteryl linoleate^24^ (CE-Bodipy, representing cholesteryl-ester) and for the HDL particle itself (apoA-I-Alexa647). After retraction the tip signal disappeared while the diffusing fluorescent molecules remained visible (Fig. 4B). The mobility of the transferred probes was identical to probes pre-inserted in DOPC membranes (Fig. 4C), confirming that both DiI and C-Bodipy were incorporated in the lipid bilayer. Occasionally, we observed the formation of membrane tethers during retraction (see the exemplary trace in Fig. 4A), further confirming the tight interaction of the HDL-coated tip with the supported lipid bilayer.

## Conclusions

Taken together, our data leads us to suggest that – in synthetic membranes – particle contact and incorporation into the lipid bilayer are sufficient requirements for cargo delivery. Incorporation of the lipoprotein particle into a lipid bilayer requires the accommodation of the apoA-I by the upper leaflet of the target membrane, which will be facilitated by the high mobility of the hydrophobic/hydrophilic interface of the lipid bilayer. Together with previous results on the high flexibility of ApoA-I in HDL particles^8–10^ our results suggest the following model: conformational changes of apoA-I α-helical regions lead to their dissociation from the particle surface and the loose association of parts of apoA-I with the target membrane. In a second step, incorporation of the particle into the lipid bilayer occurs.

The ability of lipoproteins to integrate into lipid membranes seems remarkable. Interestingly, the presence of blister-like structures within the lipid bilayer has been frequently postulated. For example, Khandelia *et al*. found via molecular dynamics simulations the formation of triacylglyceride complexes in the core of a lipid bilayer^25^. Similarly, the morphogenesis of lipid droplets at the ER membrane is believed to occur via the protein-induced nucleation of globules of neutral lipids within the two bilayer leaflets^26^. Here, we show via AFM for the first time directly the integration of HDL particles in lipid bilayers. It seems as if lipid membranes as mimicries of cell membranes were capable of storing hydrophobic molecules at least transiently, before further processing by the cell via uptake or degradation.

## Materials and Methods

### Reagents

Alexa Flour 647, 1,1′-dioctadecyl-3,3,3′,3′-tetramethylindocarbocyanine perchlorate (DiI) was obtained from Invitrogen. Sephadex G-25 fine resin, egg phosphatidylcholine (PC), cholesteryl oleate, sodium cyanoborohydride (NaCNBH_3_), triethylamine (TEA), 3-aminopropyl-triethoxysilan (APTES), ethanolamine (ETA), sodium deoxycholate and HEPES were from Sigma. 1,2-Dioleoyl-*sn*-glycero-3-phosphocholine (DOPC) was purchased from Avanti Polar Lipids. Cholesterol linked to boron dipyrromethene difluoride at sterol carbon-24 (cholesterol- Bodipy; C-Bodipy) was synthesized as described^23^. Cholesteryl-ester- Bodipy (CE-Bodipy) was synthesized by conjugating linoleic acid to C-Bodipy as described^24^.

### HDL Isolation and Labeling

Plasma, obtained from normolipidemic healthy volunteers, was isolated by serial ultracentrifugation^27^. Briefly, plasma was recovered from whole blood by centrifugation (twice at 3000 g, 4 °C, 20 min) and the density was adjusted to 1.063 g/L with KBr. Samples were centrifuged (52.000 RPM, rotor 55.2 Ti (Beckman Coulter), 4 °C, 20 h) and the upper phase containing VLDL and LDL was discarded. The density of the bottom fraction was adjusted to 1.21 g/L with KBr and samples were centrifuged as described above. The upper phase containing HDL was recovered and centrifuged again at a density of 1.21 g/L to ensure complete removal of albumin. HDL was finally dialyzed extensively against 0.9% NaCl and 1% EDTA, pH 7.4, to remove KBr. The protein concentration was determined by the Bradford method^28^. All experiments and analyses were conducted in accordance with Austrian laws and guidelines. Isolation of lipoproteins from healthy subjects was approved by the Ethics Committee of the Medical University of Vienna (licence #1414/2016). Informed consent was obtained from all subjects.

The apolipoproteins of HDL or reconstituted HDL particles (rHDL) were covalently labeled with Alexa647-succinimidyl ester according to the protocol (Life Technologies). Specifically, HDL or rHDL was diluted to 1 mg/ 450 µl PBS. Afterwards, 50 µl of sodium-bicarbonate (1 mol/l in a.dest) were added followed by the addition of 10 µl Alexa647-succinimidyl ester (10 mg/ml in DMSO). The reaction mixture was incubated at room temperature for 1 h and separation of labeled HDL from free dye was performed by gel filtration chromatography (Sephadex G-25 fine resin). To incorporate hydrophobic lipid surrogates (C-Bodipy, CE-Bodipy or DiI) into lipoprotein particles, reconstituted HDL particles (rHDL)^29^ were used and prepared as described previously^24^. Briefly, HDL was delipidated, dried under nitrogen, and resuspended in buffer A (150 mM NaCl, 0.01% EDTA and 10 mM Tris/HCl, pH 8.0). PC (2.8 mg), cholesterol (67 µg), and cholesteryl oleate (500 µg) were dissolved in chloroform: methanol, 2:1, mixed, and the solvents were evaporated. Cholesterol or cholesteryl oleate or PC was replaced by C-Bodipy or CE-Bodipy or DiI, respectively. Dried lipid films were resuspended in 200 µl of buffer A. Fifty µl of sodium deoxycholate (30 mg/ml in buffer A) were added to disperse the lipids and the mixture was stirred at 4 °C for 2 h. Delipidated HDL (250 µl of a 4 mg/ml suspension in buffer A) was added, and the mixture was stirred at 4 °C overnight. The estimated molar ratio of PC: cholesterol: cholesteryl oleate: protein was 100:48:22:1. Extensive dialysis was performed to separate rHDL from detergent and free dye. The cellular binding and uptake pattern of rHDL and native HDL were essentially comparable^24^. Additionally, C-Bodipy and CE-Bodipy exhibited a similar uptake distribution pattern as unlabeled cholesterol and cholesteryl-ester, respectively^24,30^. AFM experiments revealed no major size difference between nHDL and rHDL particles (Fig. 2C and S1).

### Preparation of Giant Unilamellar Vesicles (GUVs)

GUVs were prepared by electroformation^31,32^. This preparation technique produces vesicles with varying sizes from 10 to 100 µm. DOPC was dissolved in chloroform/methanol (10 mg/ml) and deposited on Pt electrodes and the solvent was evaporated by a constant N_2_ flow for 20 min. 300 µl of 100 mM sucrose was added in Lab-Tec chamber (Fisher Scientific) in a home-made chamber that allows visualization in the microscope. On the cap of this chamber we placed two holes with a distance of 5 mm for the electrodes. After the electrodes with dried lipids were incubated into the sugar solution, a voltage of 1.1 V at 10 Hz for 1 h and for another 30 min a voltage of 2.1 V at 2 Hz was applied. An AC field was applied using a function generator. The temperature used for GUV electroformation was 21 °C. For imaging we added 300 µl of a 150 mM glucose solution to the vesicle environment. Fluorescently labelled HDL was added to the suspension containing the GUV’s (final concentration of HDL 0.3 mg/ml). Images were acquired 20 min after addition of HDL by confocal microscopy.

### Preparation of Supported Lipid Bilayers

Supported lipid bilayers were formed on mica (for AFM experiments) or on glass surfaces (for combined AFM/fluorescence and for fluorescence microscopy). Mica was freshly cleaved (~1 µm thick) and glued onto glass coverslips using an optically transparent UV-glue (optical adhesive 88, Norland Products Inc.). Glass slides (d = 22 mm, Menzel) were incubated in Piranha solution (sulfuric acid: hydrogen peroxide, 3:1) for 20 min, rinsed with deionised water and ethanol and dried under nitrogen. Glass slides were then mounted onto the sample plate of the AFM/fluorescence setup. 30 µl of DOPC solution (10 mg/ml in chloroform: methanol, 3:1) was evaporated under nitrogen (20 min) and resuspended in 300 µl of PBS. For experiments on pre-inserted fluorescent lipids, we mixed the DOPC lipids with low concentrations of DiI or C-Bodipy before the evaporation step. Vesicles were prepared by sonication for 20 min and applied to a glass slide. After 20 min the bilayer had formed and glass slides were washed with PBS.

For high-speed AFM measurements only 1/10 of the incubation volume with the same lipid concentration was used.

### Tip- and Surface-Chemistry

For force spectroscopy experiments, commercially available Silicon AFM cantilevers (MSNL-10, Veeco Instruments NY) were amine-functionalized via gas-phase silanization with aminopropyl-triethoxysilane (APTES) as described^33^. A heterobifunctional linker (aldehyde-NHS) was prepared as described in^34^ but without the poly(ethylene glycol) (PEG) moiety. Then, 3.3 mg of the linker were dissolved in 0.5 ml chloroform and transferred into a small glass reaction chamber. Thirty µl of triethylamine was added, and the ethanolamine-coated AFM tips were immediately immersed for 2 h. Subsequently, the tips were washed with chloroform and dried with N_2_ gas. After rinsing with chloroform and drying, the tips were incubated for 2.5 hours in 100 µl HDL (0.06 mg/ml in PBS), to which 2 µl NaCNBH_3_ (1 M, freshly prepared in 10 mM NaOH) was added to start the reaction. Afterwards, 5 µl of ethanolamine hydrochloride (1 M, adjusted to pH 9.6) was added and incubation was continued for 10 min.

### Confocal Microscopy

GUVs were imaged with a laser scanning confocal microscope (LSM 700 AxioObserver, Zeiss). The microscope was equipped with a Plan-Apochromat 63x/1.40 Oil DIC M27 (Zeiss). The LSM 700 operates with solid-lasers (polarization-preserving single-mode fibers) at a wavelength of 639 nm and 488 nm. Signals were detected after appropriate filtering on a photomultiplier. Typically a z-stack was performed with a step size of 500 nm. Detector amplification, laser power and pinhole were kept constant for all measurements. Images were analyzed by thresholding the C-Bodipy signal; the average pixel value of signals underneath the obtained mask, which essentially follows the GUV surface, was calculated in both the C-Bodipy and the apoA-I-Alexa647 channel (see Fig. S1).

### FCS and FCCS experiments

Vesicles were incubated in glass bottom Ibidi chambers (no 1.5) for 15 minutes to settle down. Fluorescence correlation spectroscopy (FCS) and fluorescence cross-correlation spectroscopy (FCCS) measurements were done on the bottom membrane of GUVs using an Abberior Resolft Microscopy system (100X, NA1.4, Oil immersion objective, APD detectors, 488 nm and 640 nm excitation lasers). The detectors were connected to a correlator card (correlator.com) which was controlled by Flex program. Three measurements (10 seconds each) were done on each vesicle. At least 5 vesicles were measured for each sample. Obtained auto- and cross- correlation curves were fitted with a 2D or 3D diffusion plus triplet model^35^.

### High-speed AFM

High speed AFM was used as developed by the group of Ando^36,37^. It was operated in tapping mode at room temperature (25 °C) with free amplitudes of 1.5–2.5 nm and a set point of larger than 90% of the free amplitude. This technique is capable of capturing images at 30–60 ms per frame over a scan range of ~50 × 50 nm with ~100 scan lines. Importantly, the tip–sample interaction force is greatly reduced compared to conventional AFM, so that weak dynamic interactions between biological macromolecules are not significantly disturbed. In order to achieve highest spatial resolution and time resolution, special cantilevers with high resonance frequencies in liquid and low spring constants are used, which are equipped with sharp tips made via electron-beam deposition in phenol gas. For this study we used silicon nitride cantilevers (BL-AC10DS-A2, Olympus) with nominal spring constants ~100 pN/nm, a resonance frequency of ~500 kHz, and a quality factor of 2 in liquids. Measurements were performed at room temperature. Overview images with a size of 400 × 400 nm were captured at 500–1000 ms per frame. For diffusion and height analysis we reduced the image size to 50 × 50 nm and the scanning speed to 50–200 ms per frame.

### Force Spectroscopy and Bilayer Indentation Experiments

Force measurements were performed on a PicoPlus AFM (Agilent Technologies) operated under PicoView 1.6.8 (Agilent Technologies). Force distance cycles were acquired using silicon cantilevers with a spring constant of 0.01 N/m or 0.6 N/m (Veeco) at pulling velocities of 0.14–1.33 µm/sec. Commercially available silicon-nitride AFM cantilevers with silicon tips (MSNL-10) were amine-functionalized as described in Tip- and Surface-Chemistry.

The effective spring constant was determined via thermal noise analysis^38^. For Fig. 3 we show the tip-support separation Δ***z*** of an AFM tip indenting into a supported DOPC bilayer. The distance of the tip from the surface of the mica-support was calculated from ${\rm{\Delta }}z=z-{z}_{0}-F/kS$, where F denotes the force and z the cantilever position recorded during the approach curve, k the effective spring constant of the cantilever, and S the sensitivity taken from the approach curve upon bilayer penetration. The position of the mica surface ${z}_{0}$ was determined from the plateau regions observable in Fig. 3 at high forces, equivalent to full penetration of the tip through the bilayer.

F was plotted versus Δ*z* for multiple approach curves, which allows for extracting the thickness of the elastically deformed fluid bilayer, 2d, by fitting with Equation 1 ^39^:

1 $$F=\frac{\pi {\kappa }_{A}R}{4}{(\frac{2(2d-{\rm{\Delta }}z)}{{\rm{\Delta }}z})}^{2}$$

*κ*_*A*_ denotes the membrane compressibility and R the tip radius.

### Single Molecule Microscopy System

We used a home-built single molecule microscopy system for all fluorescence imaging experiments and as basis for the combination with an AFM. The system is based on a Zeiss Axiovert 200 inverted epifluorescence microscope equipped with a 100x NA=1.45 oil-immersion Plan-Apochromat TIRFM objective (Olympus). Samples were illuminated in objective-type total internal reflection (TIR) configuration via the epiport using 488 nm light from a solid state laser (Sapphire 200 mW, Coherent), 647 nm light from a Kr^+^ -laser (Innova 301, Coherent), or 532 nm light from a solid state laser (Millennia X, Spectra Physics), with intensities of 3–10 kW/cm^2^. After appropriate filtering, emitted signals were imaged on a back-illuminated, TE-cooled CCD-camera (Andor iXon Du-897 BV). For the precise control of the illumination timings we used acousto-optical modulators (1205 C, Isomet). Timing protocols were generated by an in-house program package implemented in LABVIEW (National Instruments). Illumination times were adjusted to values between 1 and 5 ms. Movies were recorded with a delay in the range of 15 to 300 ms between two consecutive images.

### Combined Single Molecule Fluorescence and Force Microscopy

A tip-scanning PicoPlus AFM (Agilent Technologies, Chandler, AZ) was mounted on the fluorescence microscope via a home-built plate on a high-precision XY-stage (Scan IM 120 × 100, Märzhäuser). For experiments, the cantilever was first aligned with the optical axis via the XY-stage, and the sample region of interest was then positioned to the field of view of the camera via x-y screws on the AFM head. The combined microscope was placed on a passive anti-vibration table without additional active damping (Newport). AFM force spectroscopy was performed using silicon cantilevers with a nominal spring constant of 0.01 N/m or 0.02 N/m (Veeco).

The two instruments were synchronized via TTL signals: the AFM was used as master, which triggered predefined imaging protocols on the fluorescence setup. Typically, we started an experiment by parking the AFM tip at a predetermined distance from the surface (~3 µm). The start of the approach curve was used to trigger the illumination protocol; this allowed for optically monitoring the approach, hold, and retraction part of the force distance cycles. Force-distance cycles were recorded with tip velocities of 3 µm/s. TIR excitation ensured that the tip was only illuminated during the short time period when it came into proximity of the bilayer, thereby limiting photobleaching.

Silicon-nitride AFM cantilevers with silicon tips (MSNL-10) were amine-functionalized as described in Tip- and Surface-Chemistry, and modified with rHDL particles. In general Si tips show no autofluorescence; however, to confirm that also the functionalized tip did not contribute to the HDL signal, we checked the fluorescence intensity of an aldehyde-functionalized tip (Fig. S4). We used commercially available silicon AFM cantilevers, which were amino-functionalized. The tip was placed on top of an artificial DOPC membrane pre-labeled with DiI to focus onto the bilayer. As the functionalized tip enters the evanescent field, it becomes visible as diffraction-limited spot. While DiI molecules show distinct mobile peaks, the aldehyde tip was not visible. We conclude that the signal of the tip was much dimmer than the signal of single fluorescent molecules. Analogous experiments were performed in the spectral channels of BODIPY and Alexa647.

### Single Molecule/Particle Tracking

For the analysis of fluorescence images, individual diffraction limited signals were selected, fitted with a Gaussian intensity profile, and tracked using in-house algorithms implemented in MATLAB (MathWorks); the single molecule positions were obtained to an accuracy of ${\sigma }_{xy}=20-40\,nm$.

In case of AFM images, topographical profiles were analyzed by cross-section profiling (we used the free software Gwyddion, and scanning and analysis software kindly provided by Toshio Ando). Individual particles were identified and fitted with a Gaussian profile, yielding the position to an accuracy of ${\sigma }_{xy}=2\,nm$. For single particle tracking, AFM images were analyzed using ImageJ.

Single molecule/particle diffusion constants were determined as described previously^40^. In brief, trajectories are characterized by a sequence of positions $\,\overrightarrow{x\,}(i)$, with i ranging from 1 to the number of observations of this trajectory. The mean square displacements *r* ^2^ were calculated as a function of the time-lag ${t}_{lag}=n({t}_{ill}+{t}_{delay})$) according to

$${r}^{2}=(\overrightarrow{x}(i)-\overrightarrow{x}(n+i)){^2}_{i=1;1+n,1+2n;\ldots }$$

with n denoting the difference in frame index. Data were analysed by fitting with Equation 2

2 $${r}^{2}=4D{t}_{lag}+4{\sigma }_{xy}^{2}$$

yielding the lateral diffusion constant D and the single molecule localization precision ${\sigma }_{xy}$.

In Fig. 4C, the density of transferred molecules upon contact of the HDL-coated tip with the bilayer was too high for single molecule tracking. We thus opted for a different strategy for estimating the diffusion constant by fitting the time-dependent dispersion of the fluorescence signal from the central source. The centre of the Gaussian distribution (x_0_, y_0_) was estimated by finding the coordinates of the brightest pixel in the image. The probability density function for 2-dimensional diffusion is given by

$$f(x,y)dxdy=\frac{1}{4\pi Dt}exp[-\frac{{(x-{x}_{0})}^{2}}{4Dt}-\frac{{(y-{y}_{0})}^{2}}{4Dt}]dxdy$$

and characterized by a variance:

$$Var={\int }_{-\infty }^{+\infty }{\int }_{-\infty }^{+\infty }[{(x-{x}_{0})}^{2}+{(y-{y}_{0})}^{2}]f(x,y)dxdy=4Dt$$

By choosing a window of appropriate size around the maximum the variance was calculated by the following equation:

$$Var=\frac{{\sum }_{x,y}[I(x,y)-b][{(x-{x}_{0})}^{2}+{(y-{y}_{0})}^{2}]}{{\sum }_{x,y}[I(x,y)-b]}$$

with (x,y) the coordinate of the respective pixel inside the estimation window, I(x,y) the brightness of the corresponding pixel and b an estimation of the overall background. b was determined by calculating the mean brightness of a five pixel ring around the estimation windows. Due to the diffusion process the 2D Gaussian function broadens over time depending on the time lag between two consecutive images and the diffusion constant. To account for this diffusional broadening, we increased the window size by several pixels for each subsequent image. Variances versus time are described by $Var=4D(t+{t}_{0})+c$, where *c* accounts for the resolution of the microscope, *t* the time measured from the first exposure after the AFM-tip was retracted from the surface, and *t*_0_ the unknown time between first contact and the first illumination after tip retraction. Rearrangement yields Equation 3

3 $$Var=4Dt+const$$

## Electronic supplementary material

Supplementary Information 

Movie S1

Movie S2
