# Supplementary material for: HDL particles incorporate into lipid bilayers – a combined AFM and single molecule fluorescence microscopy study
Source: Sci Rep. 2017 Nov 21;7:15886. doi: 10.1038/s41598-017-15949-7 (PMC5698431; doi:10.1038/s41598-017-15949-7)
Supplement: Supplementary file 1 — Supplementary Information [file 41598_2017_15949_MOESM1_ESM.pdf]

HDL particles incorporate into lipid bilayers – a combined AFM and single molecule fluorescence microscopy study

Birgit Plochberger[1,2,3], Clemens Röhrl[4], Johannes Preiner[3,5], Christian Rankl[6], Mario Brameshuber[1], Josef Madl[2], Robert Bittman[7, §], Robert Ros[8], Erdinc Sezgin[9,10], Christian Eggeling[10], Peter Hinterdorfer[2], Herbert Stangl[4,*], Gerhard J. Schütz[1,*]

Affiliations:
[1] TU Wien, Institute of Applied Physics, Vienna, 1040, Austria
[2] Johannes Kepler University Linz, Institute of Biophysics, Linz, 4020, Austria
[3] Upper Austria University of Applied Sciences, Campus Linz, Linz, 4020, Austria
[4] Medical University of Vienna, Center for Pathobiochemistry and Genetics, Institute of Medical Chemistry, Vienna, 1090, Austria
[5] Center for Advanced Bioanalysis GmbH, Linz, 4020, Austria
[6] Keysight Technologies Austria, Linz, 4020, Austria
[7] Queens College of the City University of New York, Department of Chemistry and Biochemistry, Flushing, NY 11367, USA
[8] Arizona State University, Department of Physics, Tempe, AZ 85287-1504, USA
[9] Max Planck Institute of Molecular Cell Biology and Genetics, Dresden, 01307, Germany
[10] MRC Human Immunology Unit, Weatherall Institute of Molecular Medicine, University of Oxford, Oxford, OX3 9DS, UK.

[§] deceased on 1 October 2014.

* Correspondence to:
Gerhard J. Schütz: TU Wien, Institute of Applied Physics, Getreidemarkt 9, Vienna, 1040, Austria, schuetz@iap.tuwien.ac.at, phone: +43-1-58801-13480

or

Herbert Stangl: Medical University of Vienna, Center for Pathobiochemistry and Genetics, Institute of Medical Chemistry, Währingerstrasse 10, Vienna, 1090, Austria, herbert.stangl@meduniwien.ac.at, phone +43 1 40160 38023

Supplementary Figures

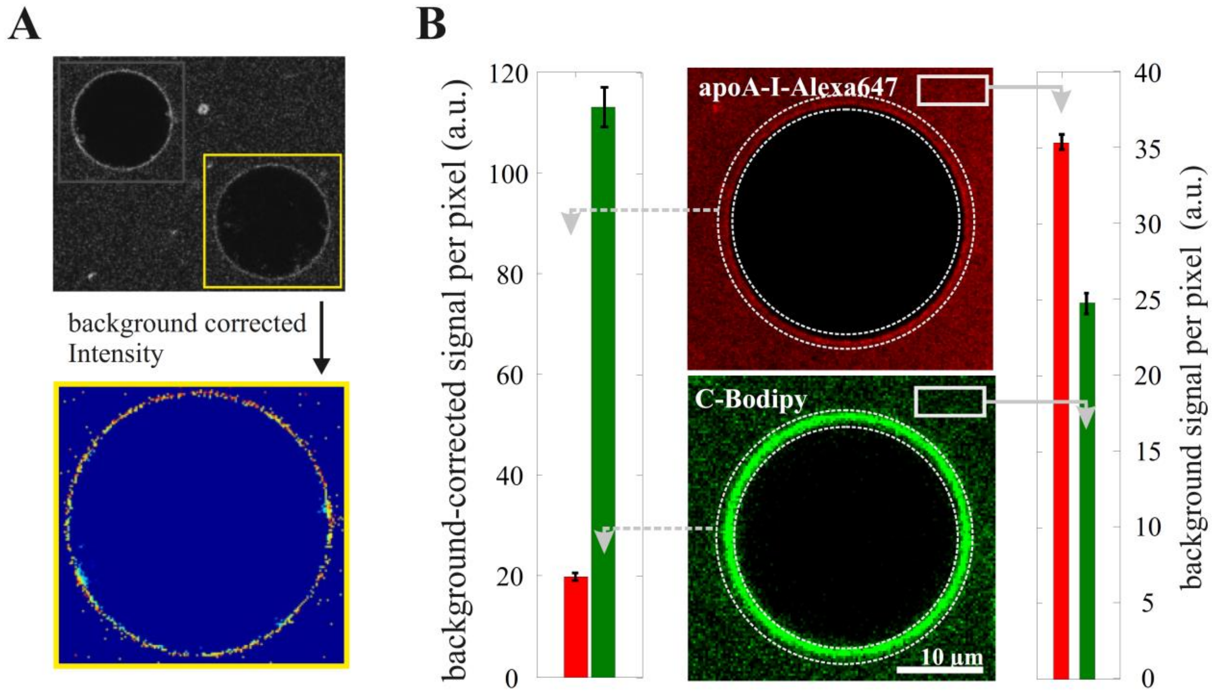

Fig. S1: Analysis of apoA-I-Alexa647 and C-Bodipy signal. (A) The top image shows a representative confocal measurement of GUVs (here shown for the apo-A-I-Alexa647 signal). The bottom image represents the signal distribution on the GUV membrane after correction. **(B)** Each color channel was corrected for background (signal outside the GUV). In a second step, the signal on the GUV membrane was averaged over the set rim.

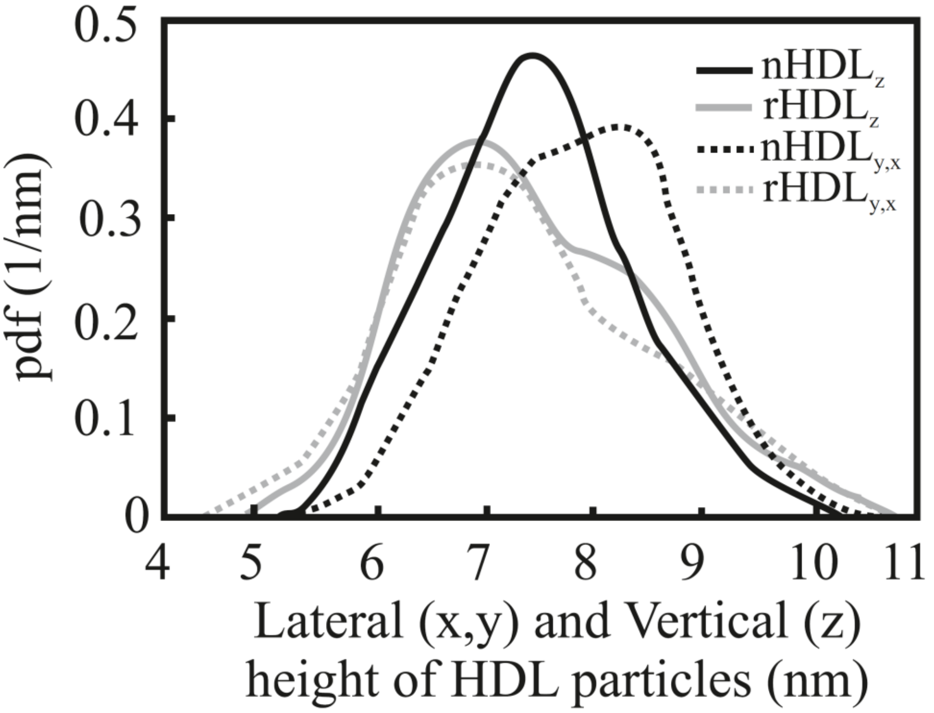

Fig. S2: Lateral and vertical size distribution of HDL Native HDL (black) and reconstituted HDL (grey) were immobilized on a mica surface. Vertical (solid lines) and lateral size (dashed lines) of individual particles was estimated by topographical imaging (n=60). The lateral size was corrected for tip convolution. Similar results were obtained for reconstituted (rHDL) and native HDL (nHDL).

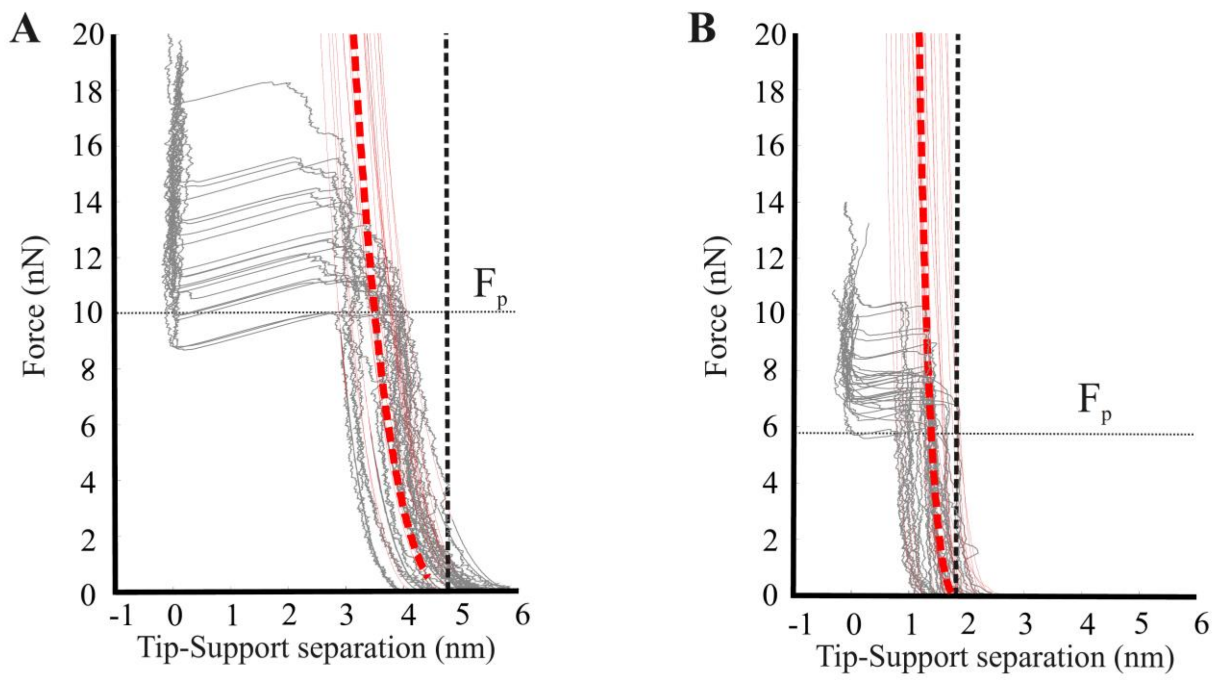

Fig. S3: AFM indentation experiments on supported lipid bilayers. Experiments were performed on mica-supported DOPC bilayers using bare silicon tips (**A**) or silicon tips covalently modified with HDL-particles (**B**). Representative approach curves (grey) are shown upon contact with the bilayer recorded with a cantilever stiffness of 0.6 N/m. The pulling velocity was varied in a range of 0.14 - 1.33 µm/s. Below the breakthrough force F_p elastic bilayer deformation can be observed, which follows the predicted behavior (red lines show fits of individual curves, red dotted line shows the averaged curve over all fits to **Eq. 1**). The intersection with the x-axis gives the thickness of the penetrated membrane layer (black dashed lines), yielding 5.2 ± 0.8 nm (**A**) and 2.0 ± 0.4 nm (**B**).

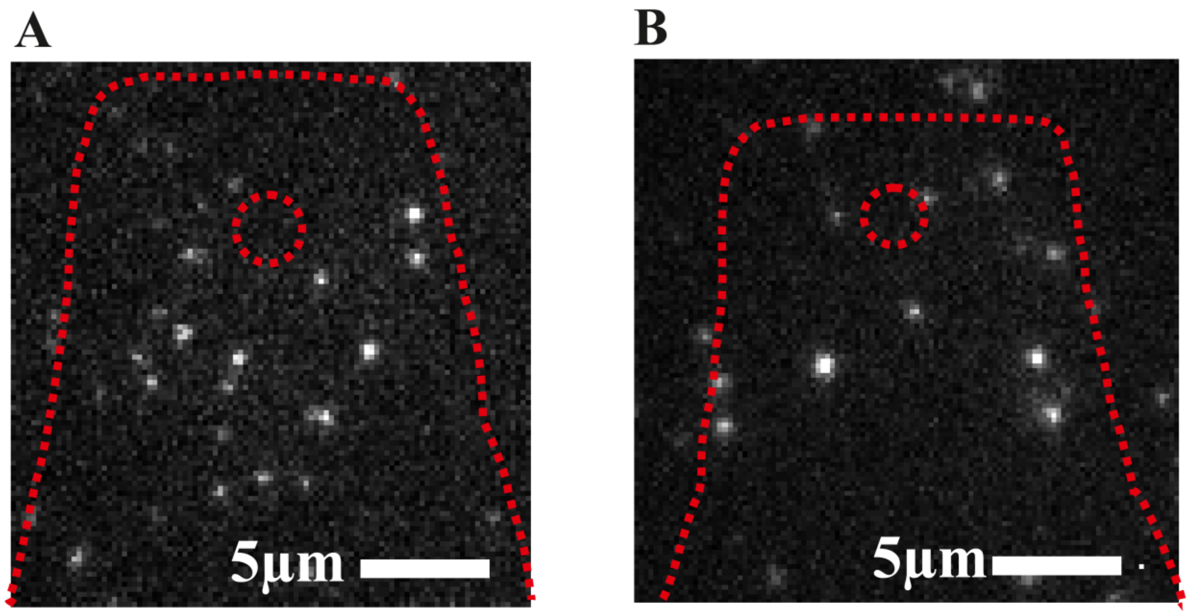

Fig. S4: Auto-fluorescence control of aldehyde-coated AFM-tips. Cantilevers were functionalized as described in the section “Tip- and Surface-Chemistry”, without protein binding. A DOPC membrane was formed on a cleaned glass slide and fluorescent lipid (DiI, DiD) was pre-inserted in order to adjust the correct focus plane. The tip was brought in contact with the membrane and fluorescence images were recorded by exciting at 532 nm (**A**) and at 647 nm (**B**). The red dotted line indicates the outline of the cantilever and the red dotted circle the position of the tip, as obtained from a transmission light image.

Supplementary Movies

Movie S1: HDL incorporates and diffuses in a synthetic lipid bilayer upon contact. Diffusion of single nHDL particles on a DOPC bilayer. The high speed AFM images were recorded with a scan size of 100 x 100 nm and a scan velocity of 90.8 ms/frame. ~60% of the observed particles showed random diffusion, while the remaining 40% were immobile. A diffusion constant D = 8.4 ± 0.45 nm^2/s was obtained for the mobile fraction. No changes were observed when we varied the AFM scanning velocity, indicating that the diffusional motion was not tip-induced.

Movie S2: **Direct observation of amphipathic cargo transfer to a lipid bilayer.** Transfer of single cargo molecules out of HDL particles. HDL was covalently linked to an AFM tip, approached to a supported DOPC bilayer, and retracted 500 ms after contact. Three experiments are shown: transfer of DiI and of C-BODIPY is clearly visible as a spread of the fluorescence signal from the contact point; CE-BODIPY was not transferred to the bilayer. Images were started during the approach of the tip to the surface, and recorded every 125 ms.
